# Relative influence of c-Kit expression and epidermal growth factor receptor gene amplification on survival in patients with non-small cell lung cancer

**DOI:** 10.3892/ol.2014.2173

**Published:** 2014-05-23

**Authors:** HUI XIAO, JUAN WANG, YANAN LIU, LI LI

**Affiliations:** 1 Department of Laboratory Medicine, Shanghai First People’s Hospital, Shanghai Jiaotong University, Shanghai 200080, P.R. China

**Keywords:** lung cancer, c-Kit, epidermal growth factor receptor, survival

## Abstract

c-Kit and epidermal growth factor receptor (EGFR) have critical roles in cell proliferation and differentiation in patients with non-small cell lung cancer (NSCLC). The present study aimed to investigate the prognostic impact of c-Kit and/or EGFR expression in tumor tissue samples from 146 patients with NSCLC. c-Kit expression was analyzed using immunohistochemistry and the expression of EGFR was assessed using fluorescence *in situ* hybridization. Univariate and multivariate analyses identified that c-Kit is a significant negative prognostic factor. The expression of c-Kit was correlated with poor differentiation, pleura involvement and smoking history (P=0.043, 0.007 and 0.032, respectively). Furthermore, patients with c-Kit-positive expression were associated with a significantly lower overall survival compared with those exhibiting c-Kit-negative expression (P=0.048). The median follow-up time was 19 months post-surgery. EGFR gene amplification as a result of polysomy of chromosome 7 was found to be negatively correlated with poor differentiation and smoking history (P=0.023 and 0.044, respectively). The findings of the present study indicate that c-Kit and EGFR expression is a strong, independent, negative prognostic factor in NSCLC.

## Introduction

Lung cancer is a leading cause of cancer-associated mortality in males and females, with a five-year survival rate of <15% (1). Increasing experimental evidence indicates that the abnormal expression of different types of receptor may influence tumor behavior and patient survival. Numerous types of tumor express the epidermal growth factor receptor (EGFR), which regulates cell proliferation, migration and differentiation (2). c-Kit is the receptor for stem cell factor and has been implicated in the pathogenesis of various types of solid tumor (3). More than 70% of cases of small cell lung cancer (SCLC) express the c-Kit receptor (4). However, little is known regarding the presence of these receptors in non-small cell lung cancer (NSCLC) and how the receptors predict patient survival.

The present study aimed to investigate whether the expression of EGFR and/or c-Kit, in tumor tissue samples from patients who had undergone surgery for NSCLC, was capable of predicting patient survival. A patient cohort of 146 subjects with NSCLC was investigated and specific immunostaining for c-Kit as well as fluorescent *in situ* hybridization (FISH) for EGFR was performed using the tumor samples. Patient survival was compared retrospectively between groups of patients expressing c-Kit and/or EGFR.

## Patients and methods

### Patients and clinical samples

The present study was approved by the Ethics Review Board of Shanghai First People’s Hospital affiliated to Shanghai Jiaotong University (Shanghai, China). Written informed consent was obtained from all patients prior to enrollment in the present study. Tumor samples from 146 patients with NSCLC who had undergone surgical resection at Shanghai First People’s Hospital between January 2009 and August 2012 were obtained. All records were anonymized in order to protect individual confidentiality. The tumor tissues were retrieved from paraffin-embedded blocks. Slides from the lung resection specimens were analyzed by two pathologists. In all cases, the diagnosis of NSCLC was established according to the 2011 World Health Organization’s classification of tumors. The clinical staging was determined according to the recommendations of the 7th International Association for the Study of Lung Cancer (5).

### Immunohistochemistry (IHC) and FISH

IHC and FISH were performed using formalin-fixed, paraffin-embedded tissue samples obtained during surgery. Sections from the tissue blocks containing >80% representative tumor tissue were used for all analyses.

For the IHC, endogenous peroxidase activity was blocked using 3% hydrogen peroxide for 20 min and the sections were de-waxed and stained with rabbit polyclonal primary antibodies against human c-Kit (phospho Y703; DakoCytomation, Glostrup, Denmark) overnight at 4°C using automatic immunostaining. The sections were incubated with reagents from a high-sensitivity detection kit (Envision detection kit; Dako, Carpinteria, CA, USA). according to the manufacturer’s instructions. Tumors were considered to be negative for c-Kit if the staining was either completely absent or observed in <5% of the neoplastic cells. Sections that were not incubated with the primary antibody served as the controls. All lung cancer cases were histologically analyzed by two pathologists and the most representative areas of the viable tumor cells were selected.

For FISH, gene copy numbers (GCNs) of EGFR per nucleus were determined using an LSI EGFR/centromeric probe for chromosome 7 (CEP 7) probe mix (Abbott Laboratories S.A., Shanghai, China). The EGFR gene was visualized as an orange signal, the CEP7 as green and the nucleus was shown as a blue signal using a 4,6-diamidini-2-phenylindone (DAPI) filter (Carl Zeiss, Oberkochen, Germany).

For FISH, 4-μm thick sections of lung cancer tissue were composed and subsequently deparaffinized, dehydrated, immersed in 0.2 N HCl and boiled in a microwave in citrate buffer (pH 6.0). The probe mixtures were added to the slides, which were incubated in a humidified atmosphere at 73°C for 5 min in order to denature the probes and target the DNA. The slides were cooled and incubated at 37°C for 19 h to allow hybridization. The slides were washed with 0.4X saline-sodium citrate (SSC)/0.3% NP-40 for 2 min at room temperature, followed by nuclear counterstaining with 2X SSC/0.1% NP-40 for 5 min at 73°C. DAPI and the anti-face compound, p-phenylenediamine were subsequently added. The signals for each probe were analyzed under a microscope equipped with a triple pass filter (DAPI/green/orange; Carl Zeiss). At least 100 tumor cell nuclei were counted per case. FISH analysis was performed independently by two pathologists, who were blinded to the patients’ clinical characteristics and to any additionally molecular variables (2). All FISH staining was performed by CSPC Ouyi Pharmaceutical Co., Ltd. (Shijiazhuang, China).

In the present study, an average value of ≥2.4 EGFR GCN signals per nucleus was determined as the cut-off for EGFR gain, although an EGFR/CEP7 ratio of ≥2.0 is the true amplification cut-off, by counting 120 cells. A Sartore-Bianchi cut-off of 2.4 GCN per nucleus was used, as it was the lowest value that was reported in previous studies. This value preserved the sensitivity and specificity of the assessments in the specific clinical setting of the present study, where only carcinomas that were identified using screening procedures and analyzed at the point of diagnosis were included. Gene amplification of EGFR as a result of polysomy of chromosome 7 was considered to be indicated by an increase in EGFR signals (≥2 signals per nucleus) as well as an increase in chromosome 7, as measured by the number of CEP7 green signals per nucleus.

### Statistical analysis

Statistical analyses were performed using SPSS 17.0 software (SPSS, Inc, Chicago, IL, USA). All of the patients were included in the statistical calculations and were followed-up until November 1, 2012. The χ^2^ test and Fisher’s exact test were used to assess the association between molecular marker expression and various clinicopathological parameters. Unadjusted survival estimates based on the c-Kit expression status were calculated using the Kaplan-Meier method and compared using the log-rank test. All tests of statistical significance were two-sided and P<0.05 was considered to indicate a statistically significant difference.

## Results

### Patient baseline characteristics

Demographic and clinical variables are shown in Table I. The median age of the patients was 63.3 years (range, 37–79 years) and the majority were male (57.5%). The NSCLC tumors comprised 68 squamous cell carcinomas and 78 adenocarcinomas. A total of 60 patients were found to be lymph node-negative, while 66 patients exhibited lymph node metastases. Due to nodal metastasis or non-radical surgical margins, 47 (32.2%) patients received postoperative radiotherapy and/or treatment with targeted agents; however, there were no evident differences in receptor expression between the patients with and without lymph node metastases.

### Expression of c-Kit and EGFR

**Figure 1:**
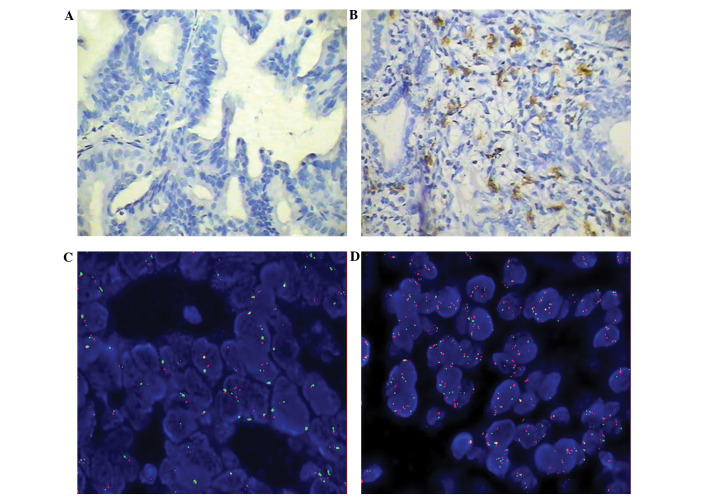
c-Kit and EGFR expression in NSCLC detected using immunohistochemistry and FISH assay. (A) c-Kit-negative NSCLC (magnification, ×200) and (B) c-Kit-positive squamous cell carcinomas cells (magnification, ×200). (C) No amplification of the EGFR and (D) amplification of the EGFR. EGFR, epidermal growth factor receptor; NSCLC, non-small cell lung cancer; FISH, fluorescence in situ hybridization.

The immunohistochemical expression of c-Kit is shown in Fig. 1, comparing representative samples of a c-Kit-negative tumor (Fig. 1A) and a c-Kit-positive tumor (Fig. 1B). In the c-Kit-positive slide, a number of cells that were positive for c-Kit were observed. The number of cells that were positive for c-Kit varied marginally among the sections, however, was calculated to be ~20% of the total cells that were visualized. The expression of c-Kit was correlated with poor tumor differentiation, pleura involvement and smoking history (P=0.043, 0.007 and 0.032, respectively; data not shown).

The GCNs of EGFR that were revealed using FISH are shown in Fig. 1C and D, which show negative and positive expression of EGFR (red) expression in tumors from different patients. In the EGFR-positive tumor sample, FISH revealed that the majority of the cells were expressing EGFR. Furthermore, EGFR gene amplification as a result of polysomy of chromosome 7 was found to be negatively correlated with poor tumor differentiation (52.9 vs. 23.5%; P=0.023; data not shown). EGFR gene amplification was not identified to differ significantly in patients with regard to gender, age, tumor location, tumor-node-metastasis stage, tumor size, lymph node metastases or involvement of the pleura. However, the absence of a history of smoking was identified to be significantly correlated with reduced EGFR gene amplification (odds ratio: 2.5) compared with a history of smoking (P=0.044).

Table II shows the distribution of c-Kit and EGFR expression in patients with NSCLC using IHC and FISH, respectively.

### Mortality

**Figure 2:**
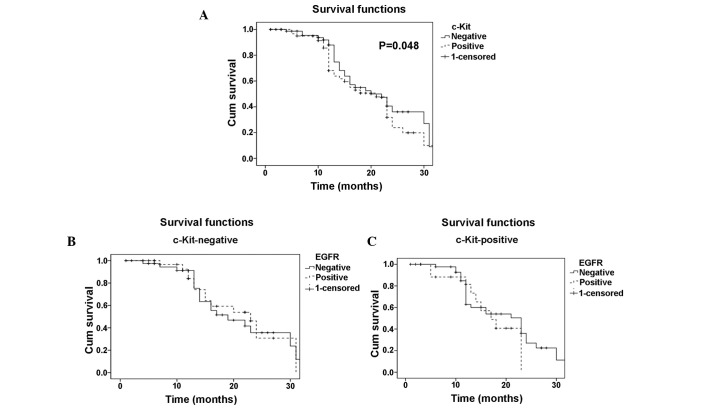
Disease-specific survival curves according to c-Kit expression. (A) Patients with c-Kit-positive expression had a lower survival rate than those with c-Kit-negative expression (median survival, 19 months vs. 24 months; P=0.048). (B) Among the c-Kit-negative patients with NSCLC, there was no significant difference in overall survival in the patients with negative EGFR expression compared with those with positive EGFR expression. (C) Among the patients with positive c-Kit expression, the patients who also exhibited a positive EGFR expression had a lower overall survival compared with those who had negative EGFR expression. EGFR, epidermal growth factor receptor; NSCLC, non-small cell lung cancer.

Fig. 2A demonstrates patient mortality between the time of surgery and 30 months post-surgery in patients with c-Kit-positive and -negative tumors. Mortality was found to be significantly higher in patients with c-Kit-positive tumors compared with those with c-Kit-negative tumors (P=0.048). In the patients with c-Kit-positive tumors, positive EGFR expression, which was detected using FISH (Fig. 2C), was observed to be associated with an increase in mortality compared with the patients with negative EGFR expression, as no patients with c-Kit- and EGFR-positive expression were found to have survived 30 months post-surgery. However, this difference was not statistically significant (P=0.06). In the patients with c-Kit-negative tumors, a similar pattern (although not significant) of increased mortality in patients with EGFR-positive tumors compared with those with EGFR-negative tumors was observed (Fig. 2B).

**Figure 3:**
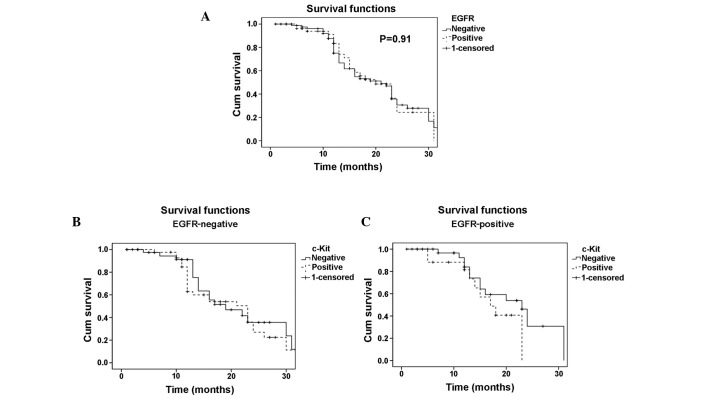
Disease-specific survival curves according to EGFR expression. (A) There was no significant difference in survival in patients with positive EGFR expression compared with those with negative EGFR expression (median survival, 15 months vs. 19 months; P=0.91). (B) Among the patients with negative EGFR expression, there was no significant difference in overall survival in the patients with negative c-Kit expression compared with those with positive c-Kit expression. (C) Among the patients with positive EGFR expression, the patients who alos had positive c-Kit expression had a lower survival than those those had negative c-Kit expression.

Fig. 3A shows the rate of mortality between the time of surgery and 30 months post-surgery in patients with EGFR-positive and -negative tumors. EGFR-negative tumors were observed to be associated with reduced mortality compared with EGFR-positive tumors, although this difference was not statistically significant (Fig. 3A; P=0.91). Furthermore, the expression of c-Kit was not found to affect mortality in patients with EGFR-negative tumors (Fig. 2B). However, in the patients with EGFR- and c-Kit-positive tumors, the rate of mortality was found to be 100% after two years, with an increased mortality rate detected in those patients with EGFR-positive tumors who were also expressing c-Kit. However, the effect of c-Kit expression in patients with varying EGFR expression was not identified to be significant (P=0.616).

After 30 months, the patients in all of the groups deteriorated and by 48 months all of the patients had succumbed (data not shown). Post-surgery therapy, where reported, did not significantly influence the effect of c-Kit and/or EGFR expression on mortality (data not shown).

## Discussion

The present study identified that certain patients with NSCLC have tumors, which express c-Kit, also known as cluster of differentiation 117, and among those patients, the GCN of EGFR varies. Mortality was found to be influenced by c-Kit and/or EGFR expression in the tumor, with patients with c-Kit-positive tumors found to have an increased mortality rate compared with those with c-Kit-negative tumors, 30 months post-surgery. Furthermore, the co-expression of c-Kit and EGFR was found to increase the rate of mortality in patients with NSCLC.

IHC for c-Kit in surgically resected NSCLC tissues revealed that patients could be divided into two subgroups based on positive or negative c-Kit expression. In addition, the GCNs of EGFR in the tumor cells was found to differ markedly between patients. These findings emphasize that NSCLC is a complex disease, which may be divided into multiple subsets, which express different proteins/genes, even though the light-microscopic histopathology may appear similar between patients. Potentially, other surface markers and gene expression profiles may divide tumors and, therefore, patients into additional subgroups, beyond grouping determined by the expression of c-Kit and the gene amplification of EGFR. However, this hypothesis is outside the scope of the present study. Other molecules which may further subdivide lung cancers include Ki67 (6), p53 (7), carcinoembryonic antigen (8), vascular endothelial growth factor (9–11), epithelial membrane antigen (11) and cytokeratin 5/6 (12), all of which have been suggested to influence disease progression and patient survival. A major problem for individualizing therapy is understanding the tumor characteristics of individual patients. The present study indicates that c-Kit and EGFR should be involved in such profiling.

One of the key findings of the present study was that the expression of c-Kit in NSCLC tumors was associated with increased mortality up to 30 months, whereas long-term survival up to 48 months was not affected. This finding is similar to reports regarding various other types of tumors, including breast cancer (13) and large cell neuroendocrine carcinoma of the lung (14), although not SCLC (15,16). Yoo *et al* (17) proposed that c-Kit expression in NSCLC did not influence survival; therefore, this requires further investigation. However, there are certain important differences between the present study, which indicates that c-Kit expression may affect patient survival, and the study by Yoo *et al* (17). The study by Yoo *et al* (17) used fewer patient samples compared with the present study and there were certain differences in the staining protocols that were used. Although such parameters may have influenced the results, further investigations are required to assess the effect of c-Kit expression on mortality in patients with NSCLC.

In the present study, the EGFR GCN in single NSLC cells was found to significantly differ between patients, furthermore, it significantly influenced mortality, albeit not as strongly as c-Kit. This finding is consistent with a study by Tsao *et al* (18), who found that EGFR gene amplification as a result of polysomy of chromosome 7, which was analyzed using FISH, was assocaited with mortality. A previous study proposed that EGFR expression influences survival in NSCLC (19), however, the study was significantly smaller than the present study and survival favored EGFR-positive patients who received concurrent therapy. Thus, the present study significantly strengthens the conclusion that EGFR GCNs do not influence disease progression and mortality. Survival may be significantly influenced by c-Kit and EGFR expression in patients with NSCLC.

In the present study, the expression of c-Kit was correlated with smoking. A total of 25 patients quit smoking within six months of commencing treatment for NSCLC and 33 continued to smoke. In those who stopped smoking at the time of diagnosis, the relative risk of NSCLC was 9 [confidence interval (CI), 4.2–21], whereas in those who continued to smoke, it was 23 (CI, 12–59). Thus, smoking may cause KIT and EGFR mutations; however, the underlying mechanism requires further functional investigations.

A limitation of the present study is that it was retrospective, and the cancer treatment was not controlled on an individual basis. However, an advantage is that the present study was relatively large compared with previous studies (20). In the present study, the overall survival beyond two years was higher than in previous studies in other institutions and countries, which is difficult to explain. Patient diagnostic criteria and/or the disease stage may be different in the previous studies. Another limitation of the present study is that it does not address other potential predictive EGFR pathway biomarkers, including EGFR mutations or functional EGFR protein expression. Of note, c-Kit positivity in NSCLC has been reported to have the potential to predict responsiveness to its inhibitor, imatinib, as this drug has been shown to be ineffective in SCLC (21,22).

In conclusion, the present study showed that c-Kit is a strong and independent prognostic factor in NSCLC, and the prognostic impact is highly associated with poor differentiation, pleura involvement and smoking history. Furthermore, mortality was observed to be significantly higher in patients with c-Kit-positive tumors. In addition, the present study demonstrated that the expression of EGFR was an independent negative prognostic factor, which is associated with differentiation and smoking history; however, patient survival was not identified to be influenced by EGFR GCN. Although this study was limited by its retrospective nature, we predict that this study will aid the establishment of novel molecular targeted therapies in the field of lung cancer.

## Figures and Tables

**Table I:** Clinicopathological characteristics of patients with non-small cell lung cancer who underwent immunohistochemical analysis to detect c-Kit expression and FISH analysis to detect EGFR expression.

	c-Kit		EGFR	
		
Characteristic	−	+	−	+
Total patients (n=146)	78	68	88	58
Gender				
Male (n=84)	45	39	52	32
Female (n=62)	33	29	36	26
P-value	0.319	-	0.416	-
Age (years)				
≤65 (n=60)	27	33	25	35
>65 (n=86)	51	35	63	23
P-value	0.806	-	0.223	-
Tumor size (cm)				
≤3 (n=70)	40	30	40	30
>3 (n=76)	38	38	48	28
P-value	0.501	-	0.187	-
Differentiation				
Well (n=40)	20	20	17	23
Moderate (n=61)	33	28	40	21
Poor (n=45)	25	20	31	14
P-value	0.043	-	0.023	-
Histology				
SCC (n=68)	37	31	42	26
Adenocarcinoma (n=78)	41	37	46	32
P-value	0.280	-	0.632	-
Pathological stage				
I (n=50)	27	23	32	18
II (n=49)	23	26	26	23
III (n=36)	25	11	24	12
IV (n=11)	3	8	6	5
P-value	0.923		0.223	
Nodal status				
No (n=80)	40	40	52	28
Yes (n=66)	38	28	36	30
P-value	0.418	-	0.069	-
Vascular infiltration				
No (n=90)	51	39	54	36
Yes (n=56)	27	29	34	22
P-value	0.516	-	0.176	-
Nerve infiltration				
No (n=93)	45	48	58	35
Yes (n=53)	33	20	30	23
P-value	0.658	-	0.138	-
Smoking history				
Never (n=41)	19	22	29	12
Current (n=32)	19	13	16	16
Former (n=73)	50	23	43	30
P-value	0.032	-	0.044	-
Involving the pleura				
No (n=100)	53	47	54	46
Yes (n=46)	25	21	34	12
P-value	0.007	-	0.150	-
TNM				
T1–T2 (n=124)	74	50	76	48
T3–T4 (n=22)	4	18	12	10
P-value	0.416	-	0.480	-

P-value is for the comparison between the parameters presented under each subheading. P<0.05 was considered to indicate a statistically significant difference. FISH, fluorescence *in situ* hybridization; EGFR, epidermal growth factor receptor; −, negative expression; +, positive expression; SCC, squamous cell carcinoma; TNM, tumor-node-metastasis.

**Table II:** c-Kit and EGFR distribution in patients with NSCLC.

	EGFR, n (%)		
		
c-Kit	−	+	Total, n
−	42 (28.76)	36 (24.65)	78
+	46 (31.51)	22 (15.07)	68
Total	88	58	146

−, negative expression; +, positive expression; EGFR, epidermal growth factor receptor; NSCLC, non-small cell lung cancer.
